# Sex Differences in the Histopathology of Acute Type A Aortic Dissections

**DOI:** 10.1055/a-2239-1741

**Published:** 2024-02-12

**Authors:** Nimrat Grewal, Onur Baris Dolmaci, Robert JM Klautz, Robert E. Poelmann

**Affiliations:** 1 Department of Cardiothoracic Surgery, Amsterdam UMC Locatie AMC, Amsterdam, Noord-Holland, Netherlands; 2 Department of Cardiothoracic Surgery, Leiden Universitair Medisch Centrum, Leiden, Netherlands; 3 Animal Sciences and Health, Leiden University, Leiden, Netherlands

**Keywords:** aorta, dissection, sex differences, histopathology

## Abstract

**Background** Although sex-related differences in cardiovascular surgery outcomes have increasingly garnered attention in the past decades, knowledge about sex disparities in the pathophysiology of acute type A aortic dissections (ATAADs) remains sparse. In this study, we evaluate the histopathologic and atherosclerotic lesions in female and male ATAAD patients.

**Methods** A total of 68 patients were studied: 51 ATAAD patients (mean age: 62.5 ± 10.8 years; 49% women) and 17 control patients (mean age: 63 ± 5.5 years; 53% women). Cardiovascular risk factors were assessed clinically. Intimal and medial histopathological features were systematically evaluated in all.

**Results** Compared to the control group, all ATAAD patients showed significantly more elastic fiber pathology, mucoid extracellular matrix accumulation, smooth muscle cell nuclei loss, and overall medial degeneration ( *p* < 0.0001). The tunica intima was significantly thinner in the ATAAD patients than in the control group ( *p* < 0.023), with the latter exhibiting significantly more progressive atherosclerotic lesions than the former. No difference in medial vessel wall pathology was seen between female and male patients. As compared to male ATAAD patients, atherosclerotic lesions were more severe in female ATAAD patients, independent of age and the cardiovascular risk factor hypertension.

**Conclusion** All ATAAD patients had a significantly thinner tunica intima and significantly diseased tunica media compared to the control patients. Our results suggest that the severity of medial aortic pathology is not sex specific in ATAAD patients. Intimal differences between females and males could, however, be considered a potential risk factor for the development of an aortic dissection.

## Introduction

An acute type A aortic dissection (ATAAD) occurs when an injury to the intima allows blood to flow between the vessel wall layers forcing the innermost layer to split from the media. An ATAAD has an incidence of 2.1 to 16.4 per 100,000 person-years, 1 making it a more common medical emergency than a ruptured abdominal aortic aneurysm. 2 An acute dissection is highly lethal if not recognized and treated aggressively. Mortality in an ATAAD is as high as 94 to 100% without treatment, with an estimated 1% per hour in the acute setting. 3 Despite advances in diagnostics and treatment strategies, mortality and morbidity of treated patients have failed to decrease in recent years. Vigorous prevention strategies are therefore urgently required. To date, the only way to prevent an acute aortic syndrome is with prophylactic aortic replacement based on screening of the aortic diameter. 4 5 At present, the guidelines do not advice sex-specific strategies in the risk stratification or treatment of an aortic aneurysms, even though women are three times more vulnerable to an acute aortic complication as compared to men and have a worse clinical outcome. 6 Moreover, most acute aortopathy occurs at a smaller aneurysm size in females than in males, even after correcting for aneurysm size to body size. 6 7

To comprehend the higher risk of aortopathy in female patients, there is a critical need to understand sex disparities in ATAAD behavior and biology. Although sex-related differences in cardiovascular surgery have in general been investigated in the past decades, knowledge about the impact of sex on the histopathological differences in the dissected aorta is poor. This study aims to identify the impact of sex differences on the histopathology of an ATAAD, which could aid in sex-specific strategies for aneurysm surveillance and treatment. We systematically describe the histopathological features of the ascending aortic wall in female and male ATAAD patients.

## Material and Methods

### Ethical Statement

Approval for the study was granted by the Medial Ethical Committee of Leiden University Medical Center (LUMC), Leiden-Den Haag-Delft (METC-LDD, ref: B21.051/MS/ms) and was conducted in accordance with the Declaration of Helsinki. Written informed consent was obtained from all ATAAD patients.

All control specimens were obtained postmortem. Obduction and tissue collection have been performed according to the guidelines and protocols for secondary tissue use of the department of pathology of the LUMC.

### Patients and Tissue Samples

Residual ascending aortic specimen was obtained in 51 ATAAD patients undergoing an emergency aortic replacement at the LUMC in Leiden, the Netherlands. Age less than 18 years, a proven genetic disorder, or other etiologies (i.e., inflammatory, infective, congenital, trauma, and pregnancy) were considered the exclusion criteria for the study.

Control aortic specimens were obtained from diseased donors ( *n* = 17); none of the donors had a cardiac cause of death. Causes of death were cancer in 6 patients, sepsis in 4 patients, pulmonary embolism in 3 patients, bowel perforation in 2 patients, pancreatitis in 1 patient, and cardiac arrest in 1 patient, without signs of coronary artery disease on coronary angiography.

Collection of circular ascending aortic wall tissue was uniformly performed at the aortotomy site in the ATAAD and control patients.

After retrieval, aortic tissue was fixed with 4% formalin (1 day) and embedded in paraffin. Transverse sections (4 μm thick) were mounted on precoated StarFrost slides (Klinipath BV, Duiven, the Netherlands). To avoid sampling error of the aortic tissue, the complete circular specimen was sectioned and studied.

Successive sections were stained with hematoxylin and eosin (H&E), resorcin-fuchsin (RF), and Movat's pentachrome. Additional immunofluorescence staining with alpha smooth muscle actin (αSMA) was performed to identify smooth muscle cell nuclei loss. The sectioning and staining protocols have been described previously. 8

### Histological Classification of the Lesions

H&E, RF, and Movat stained sections were examined for the histopathological evaluation of all samples of each circular aortic specimen. Histomorphological grading of the sections was performed according to the consensus classification for degenerative aorta pathology. 8 9 Elastic fiber fragmentation/loss, elastic fiber thinning, elastic fiber disorganization, mucoid extracellular matrix accumulation, smooth muscle cell nuclei loss, and overall medial degeneration were described as none (0), mild (1), moderate (2), or severe (3). The tunica intima thickness was measured, defined as the distance between the endothelial cell layer and the internal elastic lamina, excluding atherosclerotic lesions.

Atherosclerotic lesions were further individually classified in each section according to the modified American Heart Association classification of atherosclerosis. 10 Across the circular ascending aortic wall tissue, the atherosclerotic lesion with the most advanced grade of atherosclerosis was called the “dominant lesion” and was analyzed for this study.

All the specimens were scored blinded to the clinical characteristics of the aortic specimen and reevaluated by an independent, experienced histopathologist, which confirmed the findings.

### Statistical Analysis

All numerical data are presented as mean ± standard deviation. Statistical differences were evaluated with the Mann–Whitney *U* test for comparison between the groups. One-, two-, and three-way analysis of covariance (ANCOVA), binary logistic regression, and linear regression analysis were performed to correct for age and hypertension. Significance was assumed when *p* < 0.05. SPSS 28.0 (IBM SPSS Statistics, Version 28.0; IBM Corp, Armonk, NY) was used for the statistical analyses.

## Results

### Study Population and Clinical Characteristics

Patient characteristics of the 51 included ATAAD patients are presented in Table 1 . The ATAAD group consisted of 49% females, with no significant age difference between female and male patients (mean age of female patients: 61.3 ± 9.8 years; mean age of male patients: 63.4 ± 11.9 years; *p* = 0.493).

**Table 1:** Patient characteristics

	Male	Female	OR (95% CI)	*p* -value
*N*	26	25		
Age (y)	63.4 ± 11.9	61.3 ± 9.8	0.98 (0.93−1.03)	0.493
BAV	0	0	–	–
BMI	24.9 ± 2.4	27.3 ± 5.6	1.15 (0.98−1.36)	0.082
Hypertension	7 (26.9)	17 (68)	5.77 (1.73−19.3)	0.005
Diabetes mellitus	0	0	–	–
PAOD	0	0	–	–
COPD	0	1 (4)	1.04 (0.96−1.13)	1.000

Abbreviations: BAV, bicuspid aortic valve; BMI, body mass index; CI, confidence interval; COPD, chronic obstructive pulmonary disease; OR, odds ratio; PAOD, peripheral arterial occlusive disease.

Note: Data are presented as *n* (%), mean ± SD (standard deviation), or median (interquartile range).

The control group ( *n* = 17) consisted of 53% female patients, with no significant age difference between female and male patients (mean age of female patients: 60.4 ± 6.1 years; mean age of male patients: 63.2 ± 5.4 years; *p* = 0.73).

All included patients had a tricuspid aortic valve, and no underlying aortic genetic disease was identified after genetic screening by the institutional geneticist ( Table 1 ).

The mean body mass index (BMI) of all ATAAD patients was 26 kg/m ^2^ ; 19 patients (37%) were considered overweight (BMI > 25 kg/m ^2^ ), with no significant differences in BMI between female and male patients ( Table 1 ). The majority of ATAAD patients had a history of hypertension (59%), defined as antihypertensive medication or systolic blood pressure greater than 140 mm Hg and diastolic blood pressure greater than 90 mm Hg in the period. Incidence of hypertension was significantly higher in female than in male ATAAD patients ( *p* < 0.005). None of the ATAAD patients had a history of diabetes or peripheral arterial disease. The smoking status was not known for most patients.

In control patients, data on cardiovascular risk factors could not be retrieved for all.

### Ascending Aortic Wall Pathology

**Fig. 1:**
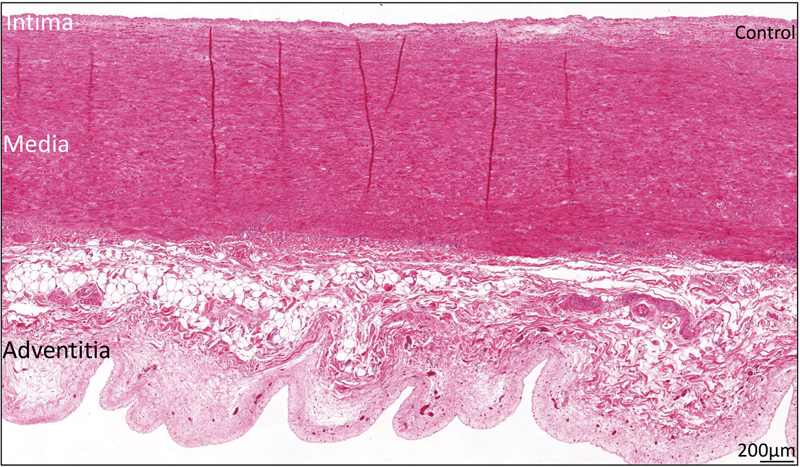
Transverse histologic section of the ascending aortic wall in a control patient, stained with hematoxylin and eosin. The intimal, medial, and adventitial layers are annotated. The scale bar is shown in the figure.

**Fig. 2:**
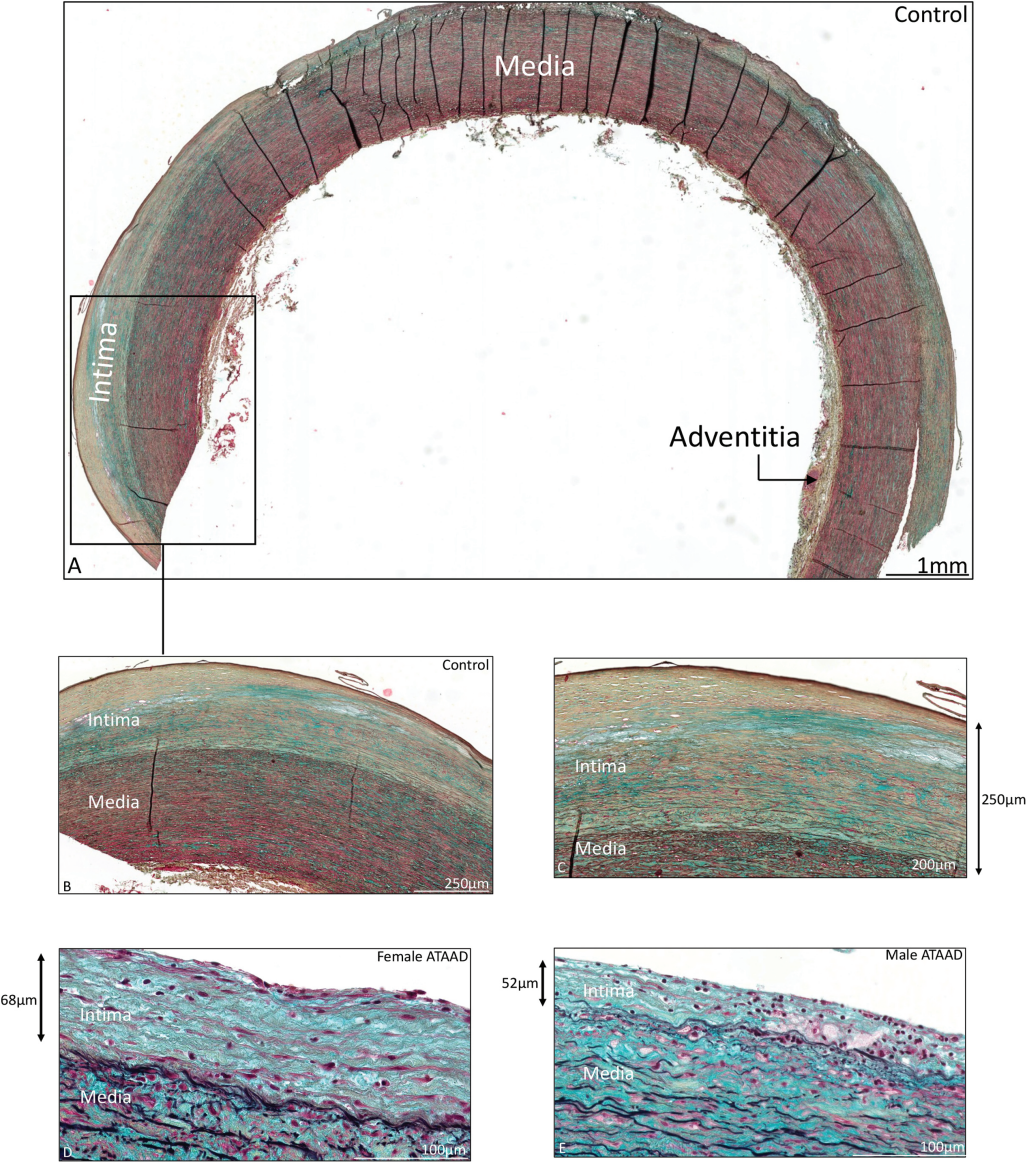
Transverse histologic section of ascending aortic wall specimen in three patients: control patient **(A–C)** ; female type A aortic dissection patient **(D)** ; male type A aortic dissection **(E)** . **(A)** An overview of the ascending aortic wall in a control patient containing three layers, which are indicated in the intima, media, and adventitia panels. A detail view of Fig. 2A is shown in Fig. 2B and C. The intimal and medial layer are labeled in figure subparts **A–E** . The tunica intima is significantly thinner in type A aortic dissection patients **(D,E)** as compared to the control patient **(A–C)** . The thickness of the tunica intima is indicated with an *arrow* besides **C, D,** and **E** . No significant difference in tunica intima thickness was seen between female and male type A aortic dissection patients **(D,E)** . ATAAD, acute type A aortic dissection. The scale bar is shown in the figure.

Fig. 1 shows an overview of the normal ascending aortic wall. The tunica intima was significantly thinner in all ATAAD patients as compared to the control specimen (143 ± 126.5 vs. 193 ± 132 μm, *p* < 0.023; Fig. 2 ; Table 2 ). No difference in intimal thickness was noted between female and male ATAAD patients ( *p* = 0.669; Fig. 2D, E ; Table 2 ). No significant difference in intimal thickness was further observed when corrected for age or in the presence of (a history of) hypertension (age vs. intimal thickness; *p* = 0.406; hypertension vs. intimal thickness; odds ratio [OR]: 1.00 [95% confidence interval (CI): 1.00–1.00], *p* = 0.860).

**Table 2:** Histopathological features in ATAAD patients

	Male	Female	OR (95% CI)	*p* -value
*N*	26	25		
Intimal thickness (μm)*	118 (76.3–188)	91 (56.5–160)	1.001 (0.997–1.005)	0.669
**EFF/L**				
None	–	–	1.1 (0.48–2.50)	0.824
Mild	6 (23.1)	7 (28)		
Moderate	14 (53.8)	12 (48)		
Severe	4 (15.4)	6 (24)		
**EFT**				
None	–	–	0.78 (0.29–2.12)	0.629
Mild	4 (15.4)	6 (24)		
Moderate	17 (65.4)	16 (64)		
Severe	3 (11.5)	3 (12)		
**EFD**				
None	–	–	1.42 (0.60–3.35)	0.423
Mild	5 (19.2)	5 (20)		
Moderate	7 (26.9)	8 (32)		
Severe	4 (15.4)	8 (32)		
**EMD**				
None	–	–	0.95 (0.34–2.67)	0.923
Mild	2 (7.7)	1 (4)		
Moderate	7 (26.9)	12 (48)		
Severe	8 (30.8)	8 (32)		
**MEMA**				
None	–	–	1.67 (0.58–4.80)	0.343
Mild	2 (7.7)	1 (4)		
Moderate	9 (34.6)	10 (40)		
Severe	6 (23.1)	10 (40)		
**SMCNL**				
None	–	1 (4)	0.66 (0.26–1.65)	0.373
Mild	2 (7.7)	5 (20)		
Moderate	11 (42.3)	10 (40)		
Severe	4 (15.4)	5 (20)		

Abbreviations: ATAAD, acute type A aortic dissection; CI, confidence interval; elastic fiber degeneration; EFD, elastic fiber degeneration; EFF/L, elastic fiber fragmentation/loss; EFT, elastic fiber thinning; EMD, Overall medial degeneration; MEMA, mucoid extracellular matrix accumulation; OR, odds ratio; SMCNL, smooth muscle cell nuclei loss.

**Fig. 3:**
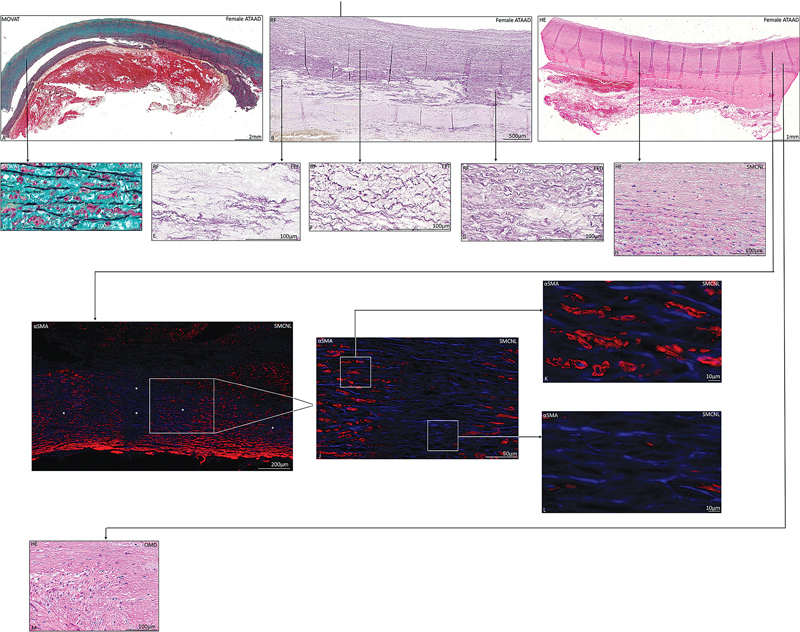
Transverse histologic section of a dissected ascending aortic wall specimen in a 69-year-old female patient **(A–M)** , stained with Movat's pentachrome **(A,D)** , resorcin-fuchsin **(B,E–G)** , hematoxylin and eosin **(C,H,M)** , and alpha smooth muscle actin **(I–L)** . The dissected ascending aortic wall is characterized by medial pathological features mucoid extracellular matrix accumulation **(A,D)** , elastic fiber fragmentation **(B,E)** , elastic fiber thinning **(B,F)** , elastic fiber disorganization **(B,G)** , smooth muscle cell nuclei loss **(C,H, I–L)** , and overall medial degeneration **(C,M)** . Immunofluorescence shows colocalization of the nuclei ( *blue* , **I** ) and αSMA ( *red* , **I–L** ) in the tunica media. Loss of smooth muscle cell nuclei is seen in panel **(I** ; areas annotated with an *asterisk* ). A detail of the medial layer is shown in panel **(J)** . An inset shows a detail of the vascular smooth muscle staining with alpha smooth muscle actin **(K)** and smooth muscle cell loss **(L)** . αSMA, alpha smooth muscle actin; ATAAD, acute type A aortic dissection; EFD, elastic fiber degeneration; EFF, elastic fiber fragmentation; EFT, elastic fiber thinning; HE, hematoxylin and eosin; OMD, overall medial degeneration; MEMA, mucoid extracellular matrix accumulation; RF, resorcin-fuchsin; SMCNL, smooth muscle cell nuclei loss. Scale bar shown in the figure.

**Fig. 4:**
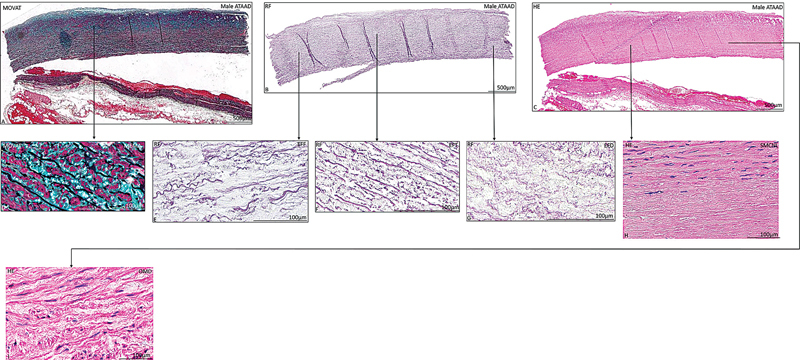
Transverse histologic section of a dissected ascending aortic wall specimen in a 72-year-old male patient **(A–I)** , stained with Movat's pentachrome **(A,D)** , resorcin-fuchsin **(B,E–G)** , hematoxylin and eosin **(C,H,I)** . No significant difference in medial pathological features was seen between the male ( Fig. 4 ) and female ( Fig. 3 ) acute type A aortic dissection patients. The dissected ascending aortic wall in the male patient is also characterized by medial pathological features mucoid extracellular matrix accumulation **(A,D)** , elastic fiber fragmentation **(B,E)** , elastic fiber thinning **(B,F)** , elastic fiber disorganization **(B,G)** , smooth muscle cell nuclei loss **(C,H)** , and overall medial degeneration **(C,I)** . The scale bar is shown in the figure. ATAAD, acute type A aortic dissection; EFD, elastic fiber degeneration; EFF, elastic fiber fragmentation; EFT, elastic fiber thinning; HE, hematoxylin and eosin; OMD, overall medial degeneration; MEMA, mucoid extracellular matrix accumulation; RF, resorcin-fuchsin; SMCNL, smooth muscle cell nuclei loss.

Medial layer pathology was significantly more profound in all ATAAD patients as compared to the control group ( Fig. 3 ; Table 2 ): mucoid extracellular matrix accumulation ( *p* > 0.0001; Fig. 3A, D ; Table 2 ); elastic fiber fragmentation and loss ( *p* > 0.0001; Table 2 ; Fig. 3B, E ); elastic fiber thinning ( *p* > 0.0001; Table 2 ; Fig. 3B, F ); elastic fiber disorganization ( *p* > 0.0001; Fig. 3B, G ; Table 2 ); smooth muscle cell nuclei loss ( *p* > 0.0001; Fig. 3C, H, I–L ; Table 2 ); and overall medial degeneration ( *p* > 0.0001; Fig. 3C, M ; Table 2 ). Loss of smooth muscle cell nuclei is seen in the aortic media ( Fig. 3C, H, I–L ). The medial layer pathology of the female and male ATAAD specimens was similar ( Figs. 3 and 4 ; Table 2 ).

### Intimal Atherosclerotic Lesions

Atherosclerotic lesions in the intima were scored according to the modified American Heart Association classification of atherosclerosis. 10 The classification categories and characterizations are provided in Table 3 .

**Table 3:** Atherosclerosis in ATAAD patients

Modified AHA classification	Characteristics	Male, *N* (%)	Female, *N* (%)	OR (95% CI)	* p* -value
** AHA classification**					
Normal intima	Defined as the distance between the endothelial cell layer and internal elastic lamella, without pathological features	4 (15.4)	3 (12)	0.99 (0.63–1.56)	0.960
** Nonprogressive atherosclerotic lesions**					
● Adaptive thickening	Accumulation of smooth muscle cells	8 (30.8)	14 (56)		
● Intimal xanthoma	Lipid and smooth muscle cell buildup	12 (46.2)	2 (8)		
** Progressive atherosclerotic lesions**					
● Pathological intimal thickening	Smooth muscle cells pooled in a proteoglycan-rich matrix with areas of extracellular lipid accumulation without necrosis	1 (3.8)	4 (16)		
● Early fibroatheroma	Early necrotic core covered by a thin fibrous cap	–	1 (4)		
● Late fibroatheroma	Necrotic core covered by a thick fibrous cap	–	1 (4)		
● Thin cap fibroatheroma	Necrotic core covered by a thin cap, infiltrated with rare smooth muscle cells	1 (3.8)	–		

Abbreviations: AHA, American Heart Association; ATAAD, acute type A aortic dissection; CI, confidence interval; OR, odds ratio.

**Fig. 5:**
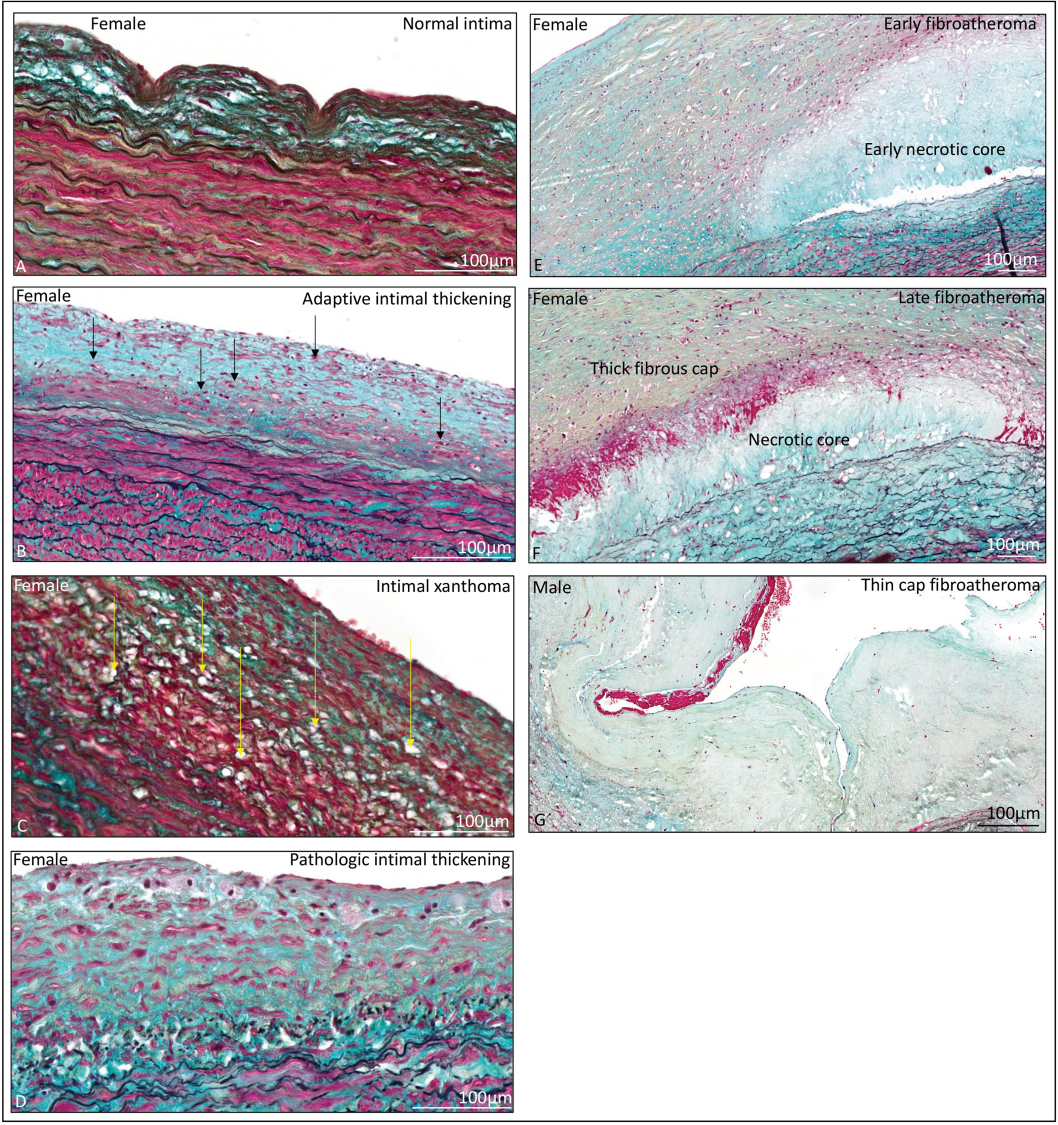
Transverse histologic section of dissected ascending aortic wall specimen obtained from female patients **(A–F)** and one male patient **(G)** . Atherosclerotic lesions are classified according to the modified American Heart Association classification. Sections are stained with Movat's pentachrome, which colors the vascular smooth muscle cells *red* , elastic fibers *dark purple* , collagen and reticulin *yellow* , and nuclei *black* . **(A)** A normal tunica intima separated from the medial layer by the internal elastic lamina. Adaptive intimal thickening **(B)** is characterized by accumulation of smooth muscle cells ( *black arrows* ). No lipid accumulation is seen in adaptive intimal thickening. Intimal xanthoma **(C)** is characterized by lipid buildup in the intimal layer ( *yellow arrows* ). Pathologic intimal thickening **(D)** develops when smooth muscle cells are pooled in a proteoglycan-rich matrix with areas of extracellular lipid accumulation without necrosis. **(E)** Early fibroatheroma in a female type A dissection patient with an early necrotic core. **(F)** In late fibroatheroma, the necrotic core is covered by a thick fibrous cap. **(G)** A thin fibrous cap is characterized by a necrotic core covered by a thin cap that is infiltrated with rare smooth muscle cells. The scale bar shown in the figure.

Seven ATAAD patients had a normal intima, 43% of which were females ( Fig. 5A , Table 3 ). Twenty-two type A aortic dissection patients demonstrated adaptive intimal thickening ( Fig. 5B , Table 3 ). In these patients, the intimal thickening consisted mainly of smooth muscle cells with an increase in proteoglycan-rich matrix. Of the patients with adaptive intimal thickening, 64% were females.

As soon as macrophage-derived foam cells become evident in the intima, the lesions are referred to as intimal xanthoma ( Fig. 5C , Table 3 ). These lesions were seen in 14 patients, of which 14% were females.

Progressive atherosclerotic lesions were seen in seven ATAAD patients ( Fi. 5D–G , Table 3 ), 86% of which were seen in female patients. Four of these seven patients demonstrated pathological intimal thickening, all of which were females ( Fig. 5D , Table 3 ). From the most severe atherosclerotic lesions, early fibroatheroma ( Fig. 5E ) and late fibroatheroma ( Fig. 5F ) were seen in female patients. Thin cap fibroatheroma was seen in a male ATAAD patient ( Fig. 5G ).

The severity of the atherosclerotic lesions was independent of age and the cardiovascular risk factor hypertension (age vs. atherosclerosis classification; *p* = 0.976; hypertension vs. atherosclerosis classification; OR: 1.14 [95%CI: 0.72–1.78]; *p* = 0.587).

In the control group, patients predominantly exhibited progressive atherosclerotic lesions. Nonprogressive atherosclerotic lesions (intimal xanthoma) were seen in only three patients. Three patients showed pathological intimal thickening, 2 had early fibroatheroma, 6 demonstrated late fibroatheroma, 1 had a healed rupture, and 2 had a fibrotic calcified plaque. Sex was randomly distributed across the nonprogressive and progressive atherosclerotic lesions in the control group.

## Discussion

A type A aortic dissection is a life-threatening condition that may be fatal if not detected and treated promptly. Current guidelines suggest prophylactic surgical aortic replacement based on a size cutoff, for both female and male individuals. However, the guidelines do not advice sex-specific strategies in the risk stratification or treatment, even though the clinical consequences are worse in women, 11 with a poorer surgical outcome and a higher mortality. 6 12 13 Despite evident clinical differences, little is known about the histopathological sex differences in ATAAD patients. Therefore, this study investigated the role of sex on the histopathology of a dissected ascending aorta in female and male patients.

Medial histopathological features were significantly distinct between the ATAAD patients and the control group, whereas the female and male ATAAD aorta did not exhibit any difference in elastic fiber pathology, mucoid extracellular matrix accumulation, medial degeneration, or smooth muscle cell nuclei loss.

Differences in intimal atherosclerosis were observed between female and male patients. Unlike abdominal aortopathy, thoracic aortopathy has not been associated with progressive aortic atherosclerosis in previous studies. 14 However, findings in earlier works might have been influenced by a male predominance in the ATAAD (study) population, with an underrepresentation of the female aortic pathology. In the current study, we sought to investigate the prevalence of atherosclerotic lesions in female and male patients according to the modified classification of the American Heart Association. 10 As both female and male patients were evenly distributed in our population with no significant difference in age, these factors could be eliminated as possible confounders of our histopathological findings. Eighty-four percent of the ATAAD group had a normal intima or exhibited nonprogressive atherosclerotic lesions. Notably, a large majority of the individuals who did develop progressive atherosclerotic lesions were females (86%, N = 6). To the best of our knowledge, this is the first study to demonstrate significant histopathological differences between male and female ATAAD patients, independent of age and cardiovascular risk factors such as hypertension. Even though medial pathological findings described in this study are not different between female and male ATAAD patients, the intimal differences could be considered as a potential risk factor for the development of an aortic dissection. As the prevalence of ascending aortic atherosclerosis in ATAADs is low, it should be highlighted that a larger study is needed to investigate the role of atherosclerosis as a potential risk stratification factor for future aortic complications in female patients. Improvement of personalized risk stratification is key to tailor treatment strategies of a life-threatening ATAAD.

## Study Limitations

Our study compared the histopathological features in the ascending aortic wall of patients with a type A aortic dissection and control specimen. Our approach is limited due to the absence of frozen tissue samples to confirm our semi-quantitively analyzed immunohistochemistry data. Our study is further limited by the relatively low number of control patients as compared to the dissected specimen. Genetic screening is performed in the patients to exclude an aortic genetic disease; however, unknown genetic mutations associated with sporadic type A dissections can be missed.
